# Not So Fast: Incomplete Assumptions of Adverse Thyroid Effects from PFAS Exposure

**DOI:** 10.1021/acs.est.5c12263

**Published:** 2025-12-02

**Authors:** Angela M. Leung, Elizabeth N. Pearce, Gregory A. Brent, Josef Köhrle

**Affiliations:** † Division of Endocrinology, Diabetes, and Metabolism, Department of Medicine, 12222 University of California Los Angeles David Geffen School of Medicine, Los Angeles, California 90095, United States; ‡ Division of Endocrinology, Diabetes, and Metabolism, Department of Medicine, Veterans Affairs Greater Los Angeles Healthcare System, Los Angeles, California 90073, United States; § Section of Endocrinology, Diabetes, Nutrition, and Weight Management, Department of Medicine, 12260 Boston University Chobanian & Avedisian School of Medicine, Boston, Massachusetts 02118, United States; ∥ 14903 Charité-Universitätsmedizin Berlin, Corporate Member of Freie Universität Berlin, Humboldt-Universität zu Berlin, and Berlin Institute of Health, Institut für Experimentelle Endokrinologie, Berlin 10117, Germany

**Keywords:** PFAS, thyroid, hypothyroidism, hyperthyroidism, environmental toxicants, toxicology

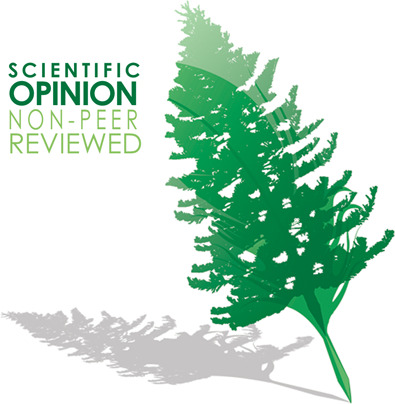

## Graphical abstract

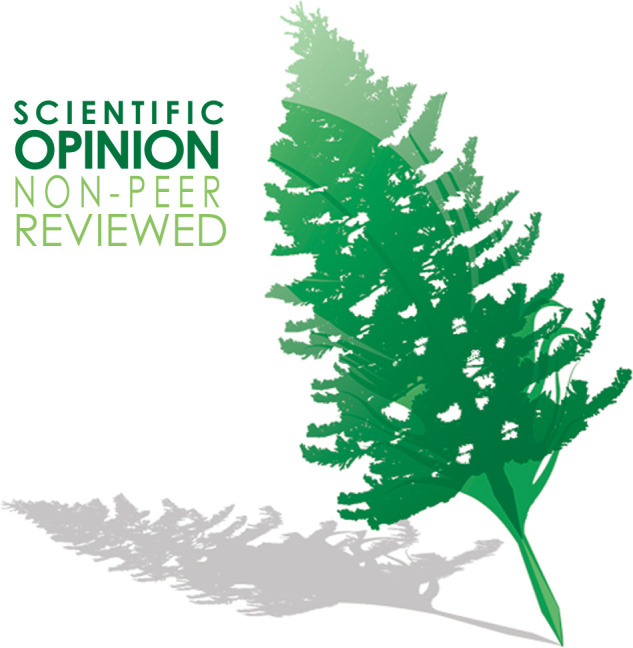

Per- and polyfluoroalkyl substances (PFAS) are a quite diverse group of more than 12 000 synthetic chemicals that have attracted substantial attention in recent decades due to their persistence in the environment and potential adverse health impacts. These toxicants share the common structure of a strong carbon–fluorine bond that results in high thermal and chemical stability, which has led to their frequent use according to their distinct functional features in a wide range of industry and consumer products manufactured since the 1940s. Commonly also termed “forever chemicals”, PFAS are resistant to breakdown and have been found ubiquitously in the environment and in biological samples from exposed humans and animals. Their stability and continuously increasing universal use for manufacturing processes and in consumer goods are leading to increased nutritional intake via drinking water and food chains, resulting in the accumulation of complex PFAS mixtures in the vast majority of humans of all ages. PFAS exposures at differing concentrations have been variably associated with health concerns, including disruption to multiple axes of the endocrine system, liver dysfunction, dyslipidemia, decreased fertility, adverse reproductive health-related outcomes, blunted vaccine effectiveness, and increased risk for cancers of the prostate, testes, kidney, and thyroid gland.

The magnitude and directionality of adverse health impacts associated with PFAS toxicity have been difficult to determine, as exposures over time are difficult to quantify, and the dose–response curves of endocrine-disrupting chemicals (EDCs) such as PFAS can be nonlinear, sigmoidal, or even more complexly nonmonotonic (i.e., no consistent increase or decrease of health risks relative to changing EDC levels).^1^ Furthermore, the foundational principles underlying hormone synthesis, distribution, metabolism, action, function, and biological concentrations may themselves be altered due to EDC interference with one or several steps of hormone synthesis, storage, distribution, cellular binding or uptake (depending on the location of their receptors), action, metabolism, and elimination. These effects may in turn influence systemic as well as local hormone availability, altering its action and disrupting the fine-tuned hormonal homeostasis and feedback regulation of the affected hormone system. The impacts of EDCs must also consider the intricate relationships between hypothalamic and pituitary regulation of hormone production from endocrine glands, physiologic hormone responses to stress, diurnal variation, and the imprecision of some hormone assays.

These considerations are particularly important when investigating the relationships between PFAS concentrations and thyroid dysfunction (classically categorized as hypo- and hyperthyroidism, characterized by the under- and overproduction of thyroid hormones, respectively). The thyroid hormones, thyroxine (T4) and thyronine (T3), which may be measured as free (unbound) and/or total (both protein-bound and unbound) concentrations, are produced by the thyroid gland in response to pituitary thyroid-stimulating hormone (TSH) and hypothalamic thyrotropin-releasing hormone (TRH). Classic endocrine feedback mechanisms are based on the dynamic effects of peripheral thyroid hormone levels on the production and secretion of TSH and TRH centrally, in order to maintain homeostasis of thyroid status at the tissue level. Thyroid hormone is needed for the regulation of body homeostasis and metabolic processes across various end organs and is particularly crucial during early life and neurodevelopment. Assessing the possible thyroidal impacts of PFAS is relevant not only at the time of individuals’ exposure but also subsequently over the course of their lives and possibly even for downstream generations.

However, our current understanding of the effects of PFAS on thyroid function is based primarily on epidemiologic studies, small human cohorts, and relatively sparse *in vitro* and animal data. Human studies include those that have assessed the associations of PFAS exposure and serum thyroid hormone levels among discrete population subgroups,^2^ including sex-specific cohorts, pregnant women, preschool children, adolescents, the elderly, individuals residing near sites with known PFAS contamination, and workers occupationally exposed to PFAS. Findings have been inconsistent, and studies have reported both positive and negative significant correlations between serum PFAS concentrations and circulating free and/or total T4, T3, and TSH. Reasons for these discrepancies include the measurement of T3, T4, and TSH from non-concurrently collected sera samples in some studies, potential altered binding of thyroid hormones PFAS to circulating distributor proteins (thus impacting the analytical procedures that determine circulating free and/or total thyroid hormone concentrations), and possibly a central hypothyroid effect from PFAS exposure (in which serum TSH levels may be inappropriately low or normal in the setting of concurrently low thyroid hormones). In addition, it is possible that PFAS might directly act as an antagonist or agonist at the level of the thyroid hormone receptor without necessarily altering circulating thyroid hormone levels. The limited *in vitro* and animal data regarding thyroid function end points have allowed for greater insight into the mechanistic effects of PFAS exposure. A study of postnatal rats showed that serum T4 levels decreased by 55% following perfluorohexanesulfonate (PFHxS) exposure, potentially as a result of the toxicant’s interaction with thyroid distributor proteins in circulation, yet there were no adverse effects on thyroid hormone action in the brain as evidenced by normal phenotyping, RNA sequencing, and quantification of radial glia cell morphology.^3^ The finding of isolated low serum free T4 levels without concomitant compensatory increased TSH levels (an entity known as hypothyroxinemia) has similarly been observed in epidemiologic and human cohort studies assessing PFAS exposure.^4^

The underlying mechanisms supporting adverse effects of thyroid dysfunction, as well as other impacts to the thyroid gland, from PFAS exposure remain poorly understood.^5^ Thyroid function abnormalities described in association with PFAS exposure may have broader adverse health impacts affecting other organ systems. However, it is also possible that serum T3, T4, and TSH concentrations outside of their reference intervals in the setting of PFAS exposure may not have detrimental health impacts. More rigorous research is needed to better understand the thyroidal impact of PFAS exposure to best inform potential regulatory actions; there are currently no data regarding thyroid hormone concentrations and appropriate biomarkers or functional end points at target sites. Finally, reported findings should take into account established endocrine principles underlying hormone synthesis, distribution, metabolism, action, function, and biological concentrations, while also considering possible physiologic reasons for the inconsistent findings reported thus far.
